# Abstract of the Proceedings of the Buffalo Medical Association

**Published:** 1875-02

**Authors:** Joseph Fowler

**Affiliations:** Secretary, pro tem.


ART. II.—*Abstract of Proceedings of the Buffalo Medical Association, held Thursday evening, December 1st*, 1874. Reported by JOSEPH FOWLER, Secretary pro tem.

The Association met at the usual time and place. The president, Dr. J. P. White, in the chair. Other members present: Drs. Rochester, Samos Phelps, J. F. Miner, Brecht, W. W. Miner, Fowler.

On motion Dr. Fowler was chosen temporary secretary.

There were present about thirty medical students in response to an invitation extended them by the president of the Association, in accordance with a vote of the Society at its last meeting. The minutes of the last meeting were read and approved.

Under the head of voluntary reports of cases worthy of special interest, Dr. J. F. Miner said he might report a case now under treatment, and one which he had watched with much interest with a view not only to draw out some valuable, instructive remarks in relation to the diagnosis and treatment of the disease, but would be glad to profit by any suggestions those present might feel disposed to make. He desired Dr. Rochester's opinion of the case, as he had given the disease more careful consideration, and as his field of observation in that direction had been more extensive than that of the speaker.

The patient was a boy, six or seven years old, formerly healthy, but recently exhibiting all the evidences of Bright's disease of the kidneys.

The urine under the field of the microscope was found to contain oil globules and casts of the uriniferous tubes. The patient had recently had diptheria of rather a mild character.

Lately his mother called my attention to a dropsical effusion.

It is difficult to determine whether he has acute disease of the kidneys, or whether the casts and oil globules are to be considered as positive evidence of the existence of organic disease. Some author says the disease rarely terminates fatally in children under ten years of age.

With reference to the treatment the doctor remarked that he had given the patient cream tartar, in dracm doses, night and morning, in an aqueous solution.

At his second visit he prescribed tinct. ferri chlo. gtt. x. every four hours, using syrup as a vehicle.

The effect of the cream tartar was to move the bowels—the anasarca disappearing under the influence of time and the remedies employed; cream tartar being the most important medicinal agent.

Dr. Rochester—Have no reason to doubt the case one of albuminuria dependent on blood poison following diphtheria.

Some writer says the poison of diphtheria and scarlatina is identical. Think this idea absurd. I am very careful in forming conclusions in cases of disease of the kidneys, and only do so after repeated microscopical and chemical examination of the urine. The uriniferous casts are very apparent where albumen is present.

A cast of the tubuli uriniferi is no evidence of organic disease of the kidney.

When oil globules are present and the fatty granules appear as if broken, giving off branches, is better evidence of structural change.

An observation of Alonzo Clark, I have found a valuable aid in making a diagnoses in this disease. Dr. Clark says when you find hyaloid casts dispersed here and there, is good evidence of an organic change in the structure of the kidney.

Hyaloid casts are better evidences of such a change than fibrinous casts.

Under the use of cream tartar and a nutritious farinaceous diet, I should expect Dr. Miner's patient to recover.

Dr. Miner observed that diphtheria has been very mild in this community of late, but was now assuming a more dangerous type.

In the case referred to, he would say that the diphtheritic exudation was still present when the anasarca appeared, and could hardly say it was a sequel of diphtheria.

It is not uncommon to find albumen in the urine following diptheria; sometimes both are present at the same time.

Dr. White—Would like to call attention to a point of therapeutics in the treatment of albuminaria, as it has received only a passing notice in the remarks made.

I would recommend the use of per-sulph. of iron as better than any other chalybeate preparation. Think Dr. Flint, Sen., endorses this plan of treatment to which I called the attention of the profession more than twenty years ago. It is well known that Simpson and others give tannin and other vegetable astringants in the albuminaria of pregnancy. If organic change has taken place, they will be of no service.

My mode of administering these astringants is in combination with glycerine. Otherwise I can fully endorse what has been said on the subject.

Dr. J. F. Miner—Some authors mention this preparation, and recommend the use of the astringents as a valuable therapeutic agent in the treatment of albuminuria. Dr. Flint, Sen., after a large experience, fails to get good results from their administration, and regards them unimportant and of little value in the treatment of that disease.

Dr. White—Why not avail ourselves of the use of the astringents and preparations of iron ?

Dr. Rochester—Houghton, of England, mentions the use of per-sulph. of iron and quinine in the treatment of diphtheria. He gave the former in combination with chlorate of potash in diphtheria. Don't think that Dr. Flint, in the recent edition of his work, condemns the employment of astringents in albuminuria. There is so much conflicting authority on the action of astringents with diuretics and cathartics that we may be led to false conclusions.

They act through the blood, constringing the capillaries, relieving them of their engorgement. Sometimes patients will take tannic acid, and get no relief. In these cases often sulphate of iron will act favorably.

Think the astringents often valuable agents in the treatment of albuminuria.

Dr. PHELPS related the case of a man who had been an invalid two or three years prior to his death.

He was 45 years of age, and by occupation a drover, often being exposed to inclement weather. His symptoms pointed to irritation of the bladder, and he had been sounded for stone.

He drank largely of water, and by some physicians was supposed to have diabetes and was treated for that disease. On making a chemical and microscopical examination of the urine, it was found to contain albumen, casts, and pus, and an entire absence of any saccharine elements, and the conclusion was that there was structural disease of the kidneys. He died in a comatosed state.

A brother, on finding his own urine to resemble that of his diseased brother in appearance, was very much alarmed, but the deposit was found to contain phosphates in abundance. The point of interest in the case was the fact of the nature of his complaint not being detected, and his disease being regarded as diabetes.

Dr. WHITE—Did you make a post mortem examination? Was there difficult micturition, and did you pass the finger in the rectum? Some of the symptoms point to the prostate as the origin and seat of the trouble.

Practitioners are in the habit of calling these diseases diabetes, especially when there is frequent micturition, although the quantity may not be increased, or when the kidneys are actively at work, eliminating a large quantity of urine.

Dr. PHELPS said he did not make a post mortem or rectal examination. The urine was muddy in appearance.

Dr. W. W. MINER in the chair.

Dr. WHITE related the following case, which he believed would be interesting to the profession because of the peculiar location of epithelial cancer, or rodent ulcer.

Epithelial cancer so often situated about the lips, when seen early we are in the habit of removing—an operation quite common in practice. The case to which I desire to call your attention, is that of a lady 50 years of age, residing in Chautauqua county.

When I first saw her, she had been suffering a considerable period of time from enlargement of the greater part of labia majora and minora, extending from the mons veneris to the posterior commissure. The parts were very sensitive, excoriation considerable, the discharge copius and offensive. Her condition was one of intense, unmitigated suffering; death would have been a desired relief.

She had been told by her attending physician that she had a cancer and must die. After seeing the case I advised the removal of the growth as a *dernier ressort*, which possibly might be followed by temporary relief, but could give her little encouragement should it be removed. She was told the dangers attending the operation, and consented that it be made, choosing to die rather than live longer in her present condition. I made a digital examination of the vagina, which could only be done while she was in a state of anæsthesia, the parts being so extremely sensitive and painful to the touch, but found no indications, either vaginal or rectal, of the disease extending in those passages. Preparatory to making the operation I gave the tinct. of the chloride of iron and chlorate of potash, a combination I often employ previous to making important operations. With the assistance of Drs. O'Brien and Brush I removed the entire lobes, and brought the parts together by means of sutures. Before the parts began to unite, dysentery developed itself, a condition quite detrimental to the success of the operation. She was sustained by means of quinine, tonics and nourishing diet. At the end of two months the dysentery had disappeared, and the wound had cicatrized kindly, except near the external urinary meatus. A catheter was introduced and allowed to remain for a considerable period.

In some months she had so far recovered as to enjoy fair health, when a newly developed kernel at the urethral orifice was removed, her health very much improving ever since, and her condition made exceedingly comfortable. She has recently been able to visit me in town, and reported herself quite free from pain and in every way more comfortable. The parts are very much disfigured, but no indications of the return of the disease are visible. Believe this form of growth can be as successfully removed from the external female organs of generation as from the lips. This patient apparently exhibited the cancerous cachexia, but it may have been exganuious—two conditions quite similar in appearance.

I have never before removed so large a mass from the external female organs of generation. Her condition was so critical that it was doubtful whether she would survive the operation.

Dr. J. F. MINER—Dr. White would have us understand the disease was malignant. It is interesting to us all, and adds much to the existing evidence of the feasability of removing these growths with the possibility of getting temporary relief, sometimes staying the progress of the disease for a number of years. Should think epithelioma on the female organs of generation not infrequent. They are usually situated on the face. In the malignant form the blood becomes contaminated, the system breaks down, and the patient dies from the constitutional effects of the disease. The question is its liability to return. Think it would be proper to operate if the parts will heal. Patients gain little in duration of life in the active forms of malignant disease.

This form of epithelioma can be removed from an outer situation and may not return in a number of years, but in the great majority of cases is liable to in time.

In scirrhus of the breast it may be a long time before it returns.

One case I remember where, with Dr. White, a scirrhus tumor of the breast was removed three times, and the patient is still living. The first operation was made six years ago. Usually these cases prove fatal in three years.

Dr. WHITE—Epithelioma is more common, whether on the lips or ganitals than true cancer in the breast. Have seen many cases where scirrhus of the breast has been removed and not returned in ten or fifteen years.

The objects to be gained by their removal is to prolong life, diminish suffering, dispose of a nasty, offensive sore, to dress and make the road to the grave smoother should it return in the latter (breast). By removing it from its external situation it is more liable to return to some other locality, more frequently selecting some internal organ, its seclusion doing way with much of the pain and offensiveness incident to an external position.

Dr. J. F. MINER—Would not operate when the glands in the axilla are enlarged, the wound would not heal so readily. When the system is broken down it hardly pays to operate.

Dr. WHITE—Often the epithelial form appears as a small scaly excrescence, often falling off and again appearing without exciting any suspicion on the part of the patient as to its true nature. It should be entirely removed when first discovered and a diagnosis made clear. It is customary for me to destroy them by the use of nitric acid put on the center of the spot by means of a glass rod till it is burned deeply, and followed with a dressing composed of zinc. oxide gr. x., morph. sulph. gr vj., glycerine ℥i.

Dr. PHELPS—I remember a man who had frozen one of his ears. It soon began to enlarge, and finally one-half of it was removed, but the wound never healed. Subsequently the remainder was amputated. Still later the patient died suddenly from hemorrhage, the disease having eaten off the external carotid artery.

Dr. W. W. MINER, who was appointed at the last meeting a committee of one to look after the matter of fuel, lights and janitor's fees, was ready to report, but owing to the small number of members present, Dr. J. F. MINER moved that the committee be continued and report at the next meeting, at which time there probably would be an increased attendance of members.

The motion prevailed, and on motion of Dr. Phelps, the meeting adjourned.

